# RELATIONSHIP OF MULTISENSORY IMPAIRMENT WITH PHYSICAL PERFORMANCE IN OLDER ADULTS IN SOMMA

**DOI:** 10.1093/geroni/igae098.0087

**Published:** 2024-12-31

**Authors:** Atalie Thompson, Tyler Mansfield, Eileen Johnson, Peggy Cawthon, Elsa Strotmeyer, Jeff Williamson, Steve Cummings, Stephen Kritchevsky

**Affiliations:** Wake Forest University School of Medicine, Winston-Salem, North Carolina, United States; University of California San Francisco, San Francisco, California, United States; Sutter Health, San Francisco, California, United States; California Pacific Medical Center, San Francisco, California, United States; University of Pittsburgh, Pittsburgh, Pennsylvania, United States; Atrium Health Wake Forest Baptist, Winston-Salem, North Carolina, United States; California Pacific Medical Center, San Francisco, California, United States; Wake Forest University School of Medicine, Wake Forest University School of Medicine, North Carolina, United States

## Abstract

There is growing evidence that various sensory and cognitive functions can impact physical performance but the relationship of multisensory impairment (MSI) and cognition with mobility in older adults is not well understood. We hypothesized that MSI would be associated with worse performance on timed physical mobility assessments, but that the association would be attenuated by controlling for tests of cognitive processing speed. Participants (N=799) were older adults (mean age 76.25+/-4.95 years; 59.82% female; 84.46% Non-Hispanic White) who completed tests of physical performance, cognitive function, and multiple sensory domains (vision, olfaction, peripheral sensation, self-reported hearing). An 8-point MSI scale was created: 1 point for moderate and 2 points for severe impairment in each sense. Separate linear regression models examined the association of MSI with 400-m gait speed, extended short physical performance battery (eSPPB), 4-square step test (FSST) and stair climb test, adjusting for demographic and clinical covariates. Each additional point on the MSI scale was associated with 0.009 m/s slower walking speed (p=0.049), 0.05 lower eSPPB score (p=0.0001), and 0.2 s longer FSST time (p=0.008), but there was no association with stair climb time(p=0.59). After adjusting for DSST and Trails B, the association of MSI with 400-m gait speed and FSST were no longer significant (p>0.05) and the association with eSPPB was partially attenuated (-0.03 units/MSI point, p=0.0037). Older adults with MSI had worse mobility function which may be partially related to cognitive processing speed. Future studies should investigate if older adults with MSI would benefit from interventions to improve mobility.

